# Acceptability and feasibility of a pilot randomized controlled trial of Narrative e-Writing Intervention (NeW-I) for parent-caregivers of children with chronic life-threatening illnesses in Singapore

**DOI:** 10.1186/s12904-022-00945-0

**Published:** 2022-04-29

**Authors:** Oindrila Dutta, Geraldine Tan-Ho, Xinyi Casuarine Low, Toh Hsiang Benny Tan, Sashikumar Ganapathy, Josip Car, Ringo Moon-Ho Ho, Chun Yan Miao, Andy Hau Yan Ho

**Affiliations:** 1 School of Social Sciences; School of Computer Science and Engineering; Lee Kong Chian School of Medicine, Nanyang Technological University, Singapore, Singapore; 2 Duke-NUS Medical School, Singapore, Singapore; 3 Club Rainbow, Singapore, Singapore; 4 Palliative Care Centre for Excellence in Research and Education, Singapore, Singapore

**Keywords:** Narrative Therapy, Psychotherapy, Paediatric Palliative Care, End-of-Life Care, Randomized Controlled Trial, Cyber-Counselling

## Abstract

**Background:**

Narrative e-Writing Intervention (NeW-I) is a novel psycho-socio-spiritual intervention which aims to bridge gaps in paediatric palliative care by providing anticipatory grief support to parent-caregivers who are looking after their child with a chronic life-threatening illness in Singapore. This is done via a therapist-facilitated smartphone app that focuses on strengths and meaning derived from parents’ caregiving journey. NeW-I is empirically informed by an international systematic review and a Singapore-based qualitative inquiry on the lived experience of parental bereavement and supported by anticipatory grief interventions literature for improving the holistic well-being for parent-caregivers of seriously ill children. NeW-I is implemented in Singapore as an open-label two-armed randomized controlled trial comprising an intervention and control group.

**Methods:**

This study examined the acceptability (via analysis of participants’ post-intervention qualitative feedback and responses to a post-intervention evaluation survey) and feasibility (via records and memos of therapists’ experience of delivering the intervention) of NeW-I among 26 intervention participants drawn from the larger trial.

**Results:**

Framework analysis of participants’ post-intervention feedback revealed four themes, namely: (i) Meaningful opportunity for reflection, (ii) Congruity with parent-caregivers’ needs, (iii) Compatibility of online narrative writing and (iv) Sustainability and enhancement recommendations. The post-intervention evaluation survey showed that participants were overall satisfied with their NeW-I experience with a large number of participants acknowledging that NeW-I had improved their spiritual well-being, hopefulness about the future and perception of social support that was available to them, as well as lessened their feelings of sadness and depression, caregiver burden and fear and anxiety about their child’s illness. The research team found it feasible to deliver the intervention in the current setting.

**Conclusion:**

NeW-I is an innovative e-health tool that could immeasurably value-add to paediatric palliative care services for Asian families in Singapore and around the world.

**Trial registration:**

ClinicalTrials.gov, ID: NCT03684382, Verified: September 2018.

## Background

Global epidemiological data indicate that there is a need to focus on improving psychosocial care for children and families living with Chronic Life-Threatening Illnesses (CLTIs). For example, CLTIs accounted for 18.1% of child and youth deaths in the United States in 2016 [1]; an estimated 40,000 children and youth were living with a life-limiting illness in England in 2010 [2]; while 801,155 children in South Africa required generalized palliative care—of which 304,441 required specialized palliative care—in a given year [3]. In Singapore, child and youth deaths caused by chronic conditions increased by 27% from 120 in 2014 to 152 in 2016 [4]. While medical technology has successfully prolonged life for children with CLTIs, the disabling nature of their conditions results in them being dependent on their caregivers (typically, parents) [2]. Parents of children with CLTIs face more challenges than parents with typically developing children [5] as they need to navigate the practical and budgetary aspects of caregiving [6], relational strains with their spouse, family members and friends [7] and unintentional neglect of their healthy children [8]. Further, caring for a child with CLTI involves frequent communications with the healthcare team which could trigger anxiety and distress if parent-caregivers do not feel engaged in making care-decisions for their child [9, 10]. Indeed, the stressors faced by parent-caregivers worsen their vulnerability to depressive symptoms and fatigue and impact quality of life [11].

Paediatric Palliative Care (PPC) aims to care for the physical, psychological, social and spiritual well-being of children facing CLTIs and their families [12]. Although several paediatric palliative interventions are in place to support families who require PPC, most such interventions reach out to families after bereavement [13]. In other words, the needs of families where a child is living with a CLTI remain unmet. This is worrying because only bereavement-support interventions that are offered after the death of a child cannot provide adequate relief from suffering for grieving families [14].

Parents facing their child’s CLTI, and potential death could benefit from psychotherapeutic interventions as soon as the prognosis is known and throughout their child’s illness trajectory [15–18]. A pre-loss intervention would facilitate parents’ transition from caregiving through mortality and bereavement, and thereby mitigate adverse grief outcomes. Globally, there are a handful of evidence-based interventions for addressing anticipatory grief in patients and families facing end-of-life; however, all of the interventions are associated with positive outcomes such as improvement in quality of life, death preparedness, spiritual well-being as well as a decrease in levels of anxiety, depression, suffering and distress [19]. A well-researched example of an anticipatory grief intervention for addressing psycho-emotional and existential distress of patients at the end of life is Dignity Therapy [20]—a brief, individualized psychotherapy that provides patients an opportunity to reflect on personally-meaningful experiences as well as speak about memorable moments and wisdom that they wish to transmit to others, which is then collated into a ‘generativity document’ and presented to the patient at the close of therapy. Family Dignity Intervention (FDI) is another anticipatory grief intervention which draws from the tenets of Dignity Therapy but is tailored to the cultural nuances of Asians facing end-of-life [21]. Briefly, this is achieved by including not just patients but also their family in the therapeutic dialogue and focusing on expression of appreciation, finding reconciliation, and passing on wisdoms for sustaining the family lineage, in addition to reflection and articulation of personally important life experiences. Evidence supports the efficacy of both Dignity Therapy [22–26] and Family Dignity Intervention [27–29] in enhancing recipients’ overall quality of life and well-being and reducing their sense of distress.

### Research gap and present study

Despite the aforementioned evidence in favour of pre-loss grief support, there is no known evidence-based research protocol or clinical intervention to address the psycho-socio-spiritual needs of parent-caregivers of children with CLTIs. Bearing in mind that Asian family caregivers may be uncomfortable with explicit emotional expression even during times of grief and loss [30], an evidence-based culturally tailored pre-loss grief support intervention for Asian parent-caregivers is of paramount importance. To bridge this gap in the delivery of psychotherapy to parent-caregivers of children with CLTIs, Narrative e-Writing Intervention (NeW-I) was developed.

### The novel Narrative e-Writing Intervention (NeW-I)

NeW-I is a novel strength-and-meaning focused and therapist-facilitated intervention which is delivered via a smartphone app. The intervention provides a platform for parent-caregivers to reflect on their experiences of caring for their child with a CLTI from a new perspective (i.e., new eye), as well as to construct a renewed sense of self-understanding and identity in the context of their experience (i.e., new I) [17]. NeW-I is empirically informed by research from the Western context including the Medical Research Council Framework for the Development and Evaluation of Complex Interventions [31, 32], the meaning-reconstruction model [33], dignity therapy [20, 34] and a qualitative systematic review of the Parental Bereavement Trajectory of child loss [16]; as well as studies from the Asian context including the narrative approach to anticipatory grief [18], family dignity intervention for holistic end-of-life care [21], and the Trauma to Transformation model of Asian parental grief and bereavement [15]. Descriptions of NeW-I’s developmental underpinnings and theoretical background as well as details about NeW-I’s trial protocol (including recruitment procedures, randomization, sample size calculations, inclusion criteria, intervention design, training of therapists and primary and secondary outcomes) have been published in another journal article [17] and PhD Dissertation [35].

Briefly, NeW-I engages parent-caregivers in four sessions of weekly structured narrative writing via a smartphone app, which is followed by a written, empathetic, and psychoeducational response from the NeW-I therapist via the app. Each of the four sessions has a unique objective, with the overarching goal of enhancing parent-caregivers’ resourcefulness in coping with their caregiving responsibilities and challenges. These objectives are achieved through parent-caregivers’ reflections and the NeW-I therapist’s affirmation and positive reframing of the following aspects: (i) Past challenges that parents have successfully coped with; (ii) sources of support within their family, social network and health-and-social care ecosystem that enhanced parents’ ability to cope with challenges; (iii) wisdoms that parents have gathered through the process of caring for their child; and (iv) the meaning that parents ascribe to their lived experience of caregiving. At the end of the four-week NeW-I journey, the therapist presents parent-caregivers with a legacy document—a compiled and edited document of participants’ narrative expression during the four weekly writing sessions which is structured in a manner that empowers participants to find strength and hope in their life story of looking after their beloved child.

### Research objectives

This study aims to assess the acceptability and feasibility of NeW-I in enhancing the psycho-socio-spiritual well-being of parent-caregivers of children with CLTIs in Singapore. The specific objectives of this study are as follows: (i) To investigate whether NeW-I is an acceptable psychotherapeutic service to parent-caregivers of children facing CLTIs in Singapore; and (ii) to examine the feasibility of delivering the NeW-I protocol to parent-caregivers of children facing CLTIs in Singapore (time taken to deliver the therapy, deviations from the therapy protocol and uncompleted therapies).

## Methods

### Research design and procedures

NeW-I was implemented in Singapore as an open-label two-armed randomized controlled trial comprising an intervention group (participants engaged in an online journaling exercise that adhered to the structured NeW-I protocol) and control group (participants engaged in an online journaling exercise that is unrelated to their child’s condition and their role as a caregiver). The present paper describes the findings of a pilot study, drawing on data from 26 intervention participants who were recruited into the larger trial. The data sources include post-intervention qualitative interview feedback and responses to a post-intervention evaluation survey—both of which were completed by the intervention group, as well as the NeW-I research team’s perceptions of intervention delivery including strengths, challenges faced, and lessons learned.

#### Participant recruitment

Participants were recruited in collaboration with leading paediatric palliative care providers in Singapore including Club Rainbow Singapore, Muscular Dystrophy Association Singapore and Rare Disorders Society Singapore between February 2019 and February 2020. Recruitment was primarily through convenience sampling such that the collaborating organizations identified potential participants from amongst their beneficiaries, introduced the study to them, and shared their contact information with the research team upon verbal consent to participate. Additionally, open recruitment was carried out via flyers posted in the offices of Club Rainbow Singapore and Muscular Dystrophy Association Singapore. Such a sampling strategy ensured maximum variation in recruitment and offered all parent-caregivers of children with CLTIs in the community an equal opportunity to participate in a potentially useful intervention.

#### Inclusion criteria

Eligible individuals were parents whose child was between the ages of 0–19 years, their child had been diagnosed with a CLTI and it could be reasonably foreseen that their child would live for at least 3 months at the time of study enrolment. The definition of a ‘child’ (age range 0–19) adopted in this study is in line with the definitions adopted by local palliative care providers and legislating bodies [36–38]. All participants could speak, read, and write in English and provide informed consent. Individuals were excluded from this study if they were suffering from severe depressive symptoms and psychological distress which was identified using two screening assessments including the Patient Health Questionnaire—9 (indicated by a score greater than 19) and Kessler Psychological Distress Scale (indicated by a score greater than 29); it was deemed that such individuals would benefit more from conventional therapy [39, 40]. Individuals who ceased to meet the inclusion criteria during their study participation (such as, due to their child’s unexpected death) were also excluded and provided with alternative resources for seeking support. Any data that had been collected until the time of their participation was retained and analysed to enable a comprehensive appraisal of the study.

#### Description of therapists

The NeW-I intervention protocol was delivered by a single therapist in the research team, who was assisted by a second therapist in delivering the control protocol during peak periods of data collection. Both therapists were trained in death education and grief counselling, had prior clinical experience in working with family caregivers in palliative settings and received close supervision and mentorship from a senior counsellor in the research team and the Principal Investigator of the study. Regular team discussions were conducted to ensure that there was consistency in the approach adopted by both therapists in delivering the research protocol.

### Ethical considerations

This study was approved by Nanyang Technological University’s Institutional Review Board (IRB-2018–07-009) and adhered to the Board’s guidelines for safeguarding participants’ identity and confidentiality.

### Data collection

#### Acceptability study

To evaluate the acceptability of NeW-I, participants were engaged in a semi-structured interview by the NeW-I therapist immediately after completing the intervention. This interview explored the following broad areas: (i) impact of the intervention on participants, (ii) aspects of the intervention that participants found to be helpful, (iii) aspects of the intervention that participants found to be unhelpful and how they could be improved, (iv) challenges encountered by participants in completing the intervention, and (v) scope for enhancing intervention usability. All interviews were audio recorded, transcribed verbatim and checked for accuracy by the research team.

In addition to participants’ qualitative feedback, they were also invited to complete a retrospective evaluation survey between July and August 2020, which aimed to understand the advantages of NeW-I engagement as self-reported by participants. Specifically, participants were invited to complete the survey via an online link that was sent to them by the research team. The survey comprised a series of statements describing their NeW-I experience and they had to indicate their agreement to each statement on a five-point rating scale ranging from Strongly Agree to Strongly Disagree. The complete list of statements included in the post-intervention evaluation survey can be seen in Table 2.

#### Feasibility study

To assess the feasibility of implementing and delivering NeW-I, the research team maintained records of the following information for each participant for the intervention and control groups: (i) time needed to respond to participants and restructure their narrative writing; (ii) deviations from the intervention protocol (if any), (iii) incomplete interventions and their reasons (if any); and (iv) NeW-I therapists’ perceptions of their own competence. NeW-I therapists also record their personal experiences of delivering the intervention, their observations of participants’ experiences and responses during and after intervention, as well as any difficult or deviant cases.

### Data analysis

All interview transcripts were imported into the NVIVO software package [41] for qualitative analysis. Framework analysis was adopted since it is more structured than other methods of qualitative analysis, the process is more explicit and informed by a-priori questions and the analysis can be easily understood by readers [42]. Such a method of analysis tends to be both deductive (arising from pre-set aims and objectives) and inductive (arising from participants' view) in approach. The framework analysis was guided by Proctor’s Taxonomy of Outcomes for Implementation Research [43], which posits that actions undertaken to implement new interventions and the impact of such actions can be examined through eight conceptually unique outcomes. In this study, the analysis was informed by the four implementation outcomes which would be relevant to the focus and breadth of this research. These include: (i) Acceptability (participants’ perceptions and attitudes towards the intervention); (ii) Adoption (participants' intentional decision to try the new and evidence-based practice); (iii) Appropriateness (participants’ beliefs about the extent to which the new intervention was relevant in their setting to address identified issues); and (iv) Sustainability (participants’ views about whether the new intervention could be maintained in the long-term). To ensure research rigor and trustworthiness of findings, the data was analysed by one research team member and presented to remaining team members during perioding meetings. Themes were finalized upon reaching inter-researcher consensus.

Participants’ responses to the post-intervention evaluation survey were downloaded using Microsoft Excel and categorically tabulated. Percentages were calculated and reported.

## Results

The sample comprised 26 parents of children with CLTIs who had been allocated to the intervention group through randomization. If a given child’s mother and father participated in the study, the group allocation of the parents was determined randomly, and both parents completed the intervention as well as the post-intervention interview and evaluation independently.

Majority of the participants were female (84.62%), married (96.15%) and of Chinese ethnicity (65.38%). Participants’ employment status varied across the sample. All but one of the participants were Singapore citizens or Permanent Residents. Further, most of the participants had a seriously ill child who was in the age group of 5 to 14 years (57.69%) and they had at least one other healthy child. The seriously ill children had varying diagnoses including cerebral palsy, epilepsy as well as renal, neuromuscular, neurodegenerative, and rare genetic diseases. Table 1 provides an overview of participants’ demographic information.

**Table 1:** Demographic characteristics of participants (*N* = 26)

	Sex	Marital status	Education level	Ethnicity	Employment status	Child’s diagnosis	Child’s age (in years)	No. of healthy children
S1	M	Mar	Bachelor’s Degree	CH	UE	Cerebral Palsy	10–14	2
S2	F	Mar	Postgraduate Degree	CH	UE	Cerebral Palsy	10–14	0
S3	F	Mar	Postgraduate Degree	CH	UE	Progressive Neurodegenerative Disease	10–14	1
S4	F	Mar	Bachelor’s Degree	CH	UE	Epilepsy	15–19	1
S5	M	Mar	Bachelor’s Degree	CH	FT	Cerebral Palsy	5–9	0
S6	F	Mar	GCE 'N' or 'O' level	CH	UE	Cerebral Palsy	5–9	0
S7	F	Mar	Polytechnic Diploma	CH	Other	Cerebral Palsy	10–14	2
S8	F	Mar	Polytechnic Diploma	Other	UE	Cerebral Palsy	10–14	1
S9	F	Mar	GCE 'N' or 'O' level	CH	Other	Cerebral Palsy	15–19	1
S10	F	Mar	Bachelor's Degree	CH	Other	Cerebral Palsy	5–9	0
S11	F	Mar	Postgraduate Degree	IN	UE	Cerebral Palsy	10–14	0
S12	F	Mar	Postgraduate Degree	CH	Other	Rare Genetic Disease	0–4	2
S13	F	Mar	Bachelor's Degree	IN	UE	Cerebral Palsy	5–9	1
S14	F	Mar	Postgraduate Degree	IN	UE	Progressive Neurodegenerative Disease	10–14	0
S15	F	Mar	Bachelor's Degree	Other	PT	Neuromuscular Disease	10–14	1
S16	F	Mar	Polytechnic Diploma	CH	Other	Renal Disease	15–19	1
S17	F	Mar	GCE 'N' or 'O' level	CH	Other	Cerebral Palsy	15–19	2
S18	F	Mar	Professional Certification	MA	Other	Progressive Neurodegenerative Disease	10–14	4
S19	F	Mar	Polytechnic Diploma	MA	FT	Rare Genetic Disease	15–19	3
S20	F	Mar	GCE 'A' Level or NITEC	CH	UE	Cerebral Palsy	5–9	2
S21	M	Mar	Bachelor's Degree	CH	UE	Rare Genetic Disease	20–24	1
S22	F	Mar	Polytechnic Diploma	MA	FT	Rare Genetic Disease	15–19	3
S23	F	Div	Did Not Complete Secondary	CH	UE	Others	0–4	2
S24	F	Mar	Bachelor's Degree	CH	FT	Cerebral Palsy	0–4	0
S25	M	Mar	Bachelor's Degree	CH	FT	Rare Genetic Disease	5–9	1
S26	F	Mar	GCE 'N' or 'O' Level	MA	UE	Renal Disease	15–19	1

*M* Male, *F* Female, *Mar* Married, *Div* Divorced, *CH* Chinese, *IN* Indian, *MA* Malay, *FT* Full Time Employed, *PT* Part Time Employed, *UE* Unemployed

### Acceptability of NeW-I

Findings provided promising evidence of participants’ acceptance of NeW-I.

#### Findings from qualitative interviews

Framework analysis of participants’ post-intervention interview transcripts revealed four key themes, which include: (1) Meaningful opportunity for reflection, (2) Congruity with parent-caregivers’ needs, (3) Compatibility of online narrative writing, and (4) Sustainability and enhancement recommendations.

#### Theme 1: Meaningful opportunity for reflection (*N* = 22)

The reflective questions in the NeW-I weekly writing sessions offered participants the opportunity to reflect on the ups and downs of their daily life as a caregiver—an experience that was both meaningful and satisfying for them.

“It gives me a chance to pen down my thoughts, my feelings... It gives me a chance to relook at the situation. Sometimes when you are in the situation, you just move through. But as I answer the [NeW-I reflective] questions, it gives me a reflection of what went well and what needs to be improved.” (S18)

Participants described NeW-I as an invitation to take a step back from their usual ‘auto-pilot’ mode of functioning and think about aspects of their caregiving journey that they had coped with well as well as aspects that necessitated further attention.

“You are so caught up with the day-to-day looking after and the caregiving duties that need to be done, you don’t have time to reflect on what has been going on for the past few years.” (S10)

Taking a bird's-eye view of their entire journey of caregiving was an empowering experience for participants because it highlighted the resources and support systems that they had gathered along the way.

“This intervention gave me the opportunity and space to look back on 11 years of caring for [son’s name], and appreciate the twists and turns, changing dynamics, and people who have come into our lives.” (S2)

Further, the opportunity to share their story helped them to feel validated in their experience as a caregiver and the struggles they faced.

“I want to write everything as thoroughly as possible because this is the journey that I’m going through. I want to be as real as possible.” (S20)

#### Theme 2: Congruity with parent-caregivers’ needs (*N* = 24)

The reflective and guiding questions that structured each week’s writing session were perceived by participants to be perceptive, since it aided them in interpreting and ascribing meaning to their experiences of caregiving.

“When I write, I ask questions to myself, like am I going to answer (it) this way or that way? So, the questions help me to be more confident and emphasize (to me) to be strong… Some of the questions help (to have) insights on life.” (S14)

In addition, the responses provided by the NeW-I therapist following each week’s writing session encouraged participants to press on and identify strategies to cope with their challenges, while simultaneously providing psychoeducation about alternative resources in the community that they could reach out to.

“There is somebody you can talk to who understands what is going on in your life… I find that the advice and suggestions given by you - like scrapbooking - I’ve never thought of it. It is something that will enhance my caregiving experience.” (S16)

“The feedback (from the therapist) is a sort of affirmation… Being affirmed gives me a lot of empowerment.” (S21)

Participants also expressed their gratitude at receiving the legacy document at the close of the intervention. They explained that this tangible record of their caregiving journey had facilitated sharing of their struggles with their family and friends through the retelling of their story of caregiving and survival.

“That document opened up my bubble. My bubble tends to be at home with (child’s name), so it opened that – I have a lot of invisible webs and links to others that I don’t naturally see on a day-to-day basis. But nonetheless their presence and their imprint are quite strongly clear.” (S2)

“When I shared the legacy document with a few of my friends, they were really touched. They were like, ‘I never knew you go through so much,’ and they never knew those parts of me. It was a very impressive journey for me.” (S11)

#### Theme 3: Compatibility of online narrative writing (*N* = 14)

Participants appreciated the flexibility of the online therapeutic platform which they could engage with during their free time and from any convenient location. They elaborated that NeW-I was more convenient than a face-to-face session with a therapist since the latter involved committing to a predefined date and time, which was challenging for parent-caregivers who juggled multiple caregiving responsibilities.

“(When) you get time, you can finish it (refers to weekly NeW-I writing sessions) … Even when (I am) with the family, I can find 20 minutes, sitting around with them also... If we have to fix up appointments, I think that (is) very difficult for caregivers.” (S14)

Further, the relative anonymity and personal space that NeW-I offered to participants made it a less intimidating channel for self-expression as compared to face-to-face conversations.

“(When) you talk to someone face-to-face, you don’t have much time to think, so when I want to put my thoughts in my writing, it feels better.” (S20)

The increased personal space and anonymity of NeW-I was helpful for participants regardless of whether they perceived themselves to be introverted or socially confident about sharing their challenges with others.

“Sometimes, certain things, we feel shy to share (it) if we (are) face-to-face (with another person). We feel it is better to stay anonymous.” (S26)

“I am very vocal, so I talk a lot about what is happening. This for me is an additional resource… But if they [parents] are not very vocal or they don’t talk much about what is happening to others then it would be a good experience for them.” (S8)

#### Theme 4: Sustainability and enhancement recommendations (*N* = 23)

Participants highlighted that it was challenging to complete their weekly writing within a single session lasting 30 min while also tending to their caregiving responsibilities. They recommended a ‘Pause’ button and a ‘Save as Draft’ feature to be added to the app to resolve this issue.

“Do have a ‘pause’ button (in the app) during the writing session of 30 minutes in case there is anything that we need to attend to urgently and we have to stop writing for a while, and a ‘save’ button so that whatever we write can be saved when we need to log out of the app." (S7)

Some participants suggested that the reflective questions in the weekly writing sessions could be rephrased for greater clarity and comprehensibility, while other participants wished that NeW-I could be available in regional languages so that parent-caregivers who were not fluent in English would also have the opportunity to seek support from the intervention platform.

“Sometimes I find the question is too short. I can’t get what kind of information you all request. Every time I log in, there is only 30 minutes and I have to really think about what I’m going to write.” (S24)

“Many caregivers don’t speak English or don’t understand English. They will be more comfortable in their own language.” (S14)

Participants further expressed that it would have been useful if their NeW-I engagement had continued beyond the four weekly sessions and reception of the legacy document. They felt that periodic follow-up sessions would provide them a platform to voice their concerns and seek professional support as they navigated the ups and downs of looking after their seriously ill child.

“You can have a follow-up… find out if there are any problems, unease, or something that they (parent-caregivers) need further suggestions on how to handle.” (S22)

Finally, one participant expressed interest in having more knowledge about the NeW-I therapist’s background and expertise. The participant shared that this would help parents to understand the difference between disclosing their caregiving challenges to the therapist versus conversing with their informal social network about issues they were facing.

“The therapist needs to share what is his or her portfolio of experiences with regards to how he/she can help. Because I can do the same. I’m not a psychologist but I can help my friend when she feels depressed. So, what’s the difference between telling you as a therapist and telling me?” (S4)

#### Findings from post-intervention evaluation survey

Findings from the qualitative interviews were corroborated by participants’ self-reported responses to the post-intervention evaluation survey. As shown in Table 2, from a sample of *N* = 21 participants who completed the evaluation survey, over 85% of participants were in agreement that NeW-I had been helpful to them, with over 52% of participants expressing that NeW-I had been as helpful as other forms of support that they received from their child’s healthcare team. Majority of the participants acknowledged that NeW-I had improved their spiritual well-being (52%; *n* = 11—rounded to nearest whole number), hopefulness about the future (57%; *n* = 12) and perception of social support that was available to them (76%; *n* = 16). In fact, improvement in perception of social support emerged as the greatest benefit that participants derived from their NeW-I experience according to the post-evaluation survey results. In addition, participants concurred that NeW-I had helped to lessen their sense of sadness and depression (62%; *n* = 13), feelings of burden due to their caregiving responsibilities (52%; *n* = 11) and feelings of fear and anxiety towards their child's illness (57%; *n* = 12). A resounding number of participants (90%; *n* = 19) acknowledged that overall, they felt satisfied with their NeW-I experience.

**Table 2:** Participants’ responses to post-intervention evaluation survey (*N* = 21)

Survey Statement	Strongly Agree		Agree		Neither Agree nor Disagree		Disagree		Strongly Disagree	
	N	%	N	%	N	%	N	%	N	%
1. NeW-I has been helpful for me	4	19.05%	14	66.67%	3	14.29%	0	0%	0	0%
2. New-I has been as helpful as other aspects of support I get from my child’s healthcare team	3	14.29%	8	38.1%	8	38.1%	2	9.52%	0	0%
3. NeW-I has improved my quality of life	1	4.76%	9	42.86%	9	42.86%	2	9.52%	0	0%
4. NeW-I has improved my spiritual well-being	2	9.52%	9	42.86%	7	33.33%	2	9.52%	1	4.76%
5. NeW-I has improved my hopefulness about the future	2	9.52%	10	47.62%	8	38.1%	0	0%	1	4.76%
6. NeW-I has lessened my sense of sadness and depression	2	9.52%	11	52.38%	6	28.57%	1	4.76%	1	4.76%
7. NeW-I has lessened my feelings of burden due to my caregiving responsibilities	1	4.76%	10	47.62%	5	23.81%	4	19.05%	1	4.76%
8. NeW-I has improved how I view the social support that is available to me	3	14.29%	13	61.9%	5	23.81%	0	0%	0	0%
9. NeW-I has lessened my feelings of fear and anxiety toward my child's illness	1	4.76%	11	52.38%	7	33.33%	1	4.76%	1	4.76%
10. NeW-I has helped me to understand my child's needs better	2	9.52%	8	38.1%	7	33.33%	3	14.29%	1	4.76%
11. NeW-I has helped me to improve my relationship with my spouse	1	4.76%	7	33.33%	11	52.38%	2	9.52%	0	0%
12. NeW-I has helped me to improve my relationship with my family members (such as my children, parents, in-laws and other relatives)	0	0%	8	38.1%	11	52.38%	1	4.76%	1	4.76%
13. NeW-I has helped me to express myself better to my child's doctors, nurses and the medical team	0	0%	9	42.86%	8	38.1%	3	14.29%	1	4.76%
14. In general, I am satisfied with NeW-I	2	9.52%	17	80.95%	2	9.52%	0	0%	0	0%

### Feasibility of NeW-I: Evaluation of resources for intervention implementation

Detailed study records and audit trails revealed that the NeW-I therapist successfully responded to participants as well as prepared and sent them their respective legacy documents within the protocol timeline (detailed descriptions of the intervention procedures and their timeline can be found in the NeW-I protocol [17]). Further, the team did not experience any challenges in terms of resources and the capabilities required to manage the intervention during the NeW-I pilot trial. Physical workspace and IT resources were supplied by the University that the research team was affiliated to. The NeW-I therapists were certified in death education and grief counselling and were experienced in working with family caregivers of patients receiving palliative care. All team members had successfully completed research integrity modules under the provisions of Nanyang Technological University’s Institutional Review Board and adhered to the Board’s guidelines for safeguarding participants’ identity and confidentiality.

The research team had adequately estimated the manpower required to develop the NeW-I platform and deliver the intervention. Specifically, one computer engineer developed the online intervention platform and maintained it throughout the study period; one full-time PhD student was responsible for data collection and entry, intervention delivery, analysis of both quantitative and qualitative data as well as drafting of research reports and presentations for dissemination of findings; one research associate provided assistance with data collection and delivery of the control protocol during peak periods of participant recruitment; one senior counsellor supervised the PhD student and research associate for quality assurance of the therapist’s responses to participants; and the Principal Investigator managed and steered the entire project.

A few minor unanticipated challenges were encountered by the research team in using the new medium of intervention delivery – a smartphone app – thereby reinforcing the need for technical proficiency to develop and deliver the intervention. Briefly, technical malfunctioning of the app in the initial months of the trial and delays in restarting the server following periodic checks impeded some participants’ timely submission of their weekly writing entries, thereby resulting in a deviation from the NeW-I protocol. These participants had to email their weekly writing entries instead of submitting it via the app. Other participants were unable to complete their writing session within the stated timeline because they had not been notified about the activation of the new writing session in a timely manner. These issues were efficiently and effectively resolved by the research team by seeking out appropriate instruction from technical experts.

Clinicians and researchers who are interested in adopting NeW-I as a therapeutic tool to advance their work with parent-caregivers of children with CLTI are advised to thoroughly assess the resources at their disposal and their team’s proficiency to manage the intervention.

## Discussion

NeW-I is a novel evidence-based pre-loss intervention for enhancing the psycho-socio-spiritual well-being of parent-caregivers of children facing CLTIs in Singapore, and this study is the first empirical examination of NeW-I’s acceptability and feasibility among its target audience. Qualitative framework analysis of participants’ post-intervention interviews showed that the ritualistic process of engaging in a weekly narrative writing about their caregiving experiences drew parent-caregivers' attention to the resources that they had at their disposal and the positive outcomes of their journey of caregiving for their seriously ill child. Participants found the medium of intervention delivery (i.e., internet-based writing) to be convenient since they could access the intervention from any location and at any time, as well as private since they could share their thoughts and feelings in a format that was less intrusive than face-to-face talk. Additionally, recommendations were obtained from participants regarding strategies that could be adopted to enhance the overall delivery of the intervention and the outcomes derived through it – including flexibility in completing the writing session, delivering the intervention in regional languages, greater clarity about the expertise of the therapist and following up with participants over a longer duration than four weeks. This positive feedback provided by participants are further ratified by their self-reported responses to the evaluation survey which indicate participants’ satisfaction with the NeW-I. Lastly, an appraisal of the resources required to deliver the intervention do not reveal any major challenges, which provides further endorsement for research and delivery of NeW-I beyond this pilot trial.

In line with previous research [44], this study provides evidence for the acceptability and feasibility of a narrative intervention in enhancing parent-caregivers’ agency to cope with the challenges of looking after their seriously ill children. Further, this research provides empirical support to previous studies [45] which posited that provision of psychotherapeutic services during the illness trajectory serves to enhance the quality of parent-caregivers’ memories of the days leading to their child’s potential end-of-life and death, strengthen their sense of resilience, hope and perceived support from their social networks, thereby safeguarding them against adverse bereavement outcomes. This study also reinforces previous findings that Asian family caregivers tend to be uncomfortable about expressing their feelings openly [46, 47]. Present authors believe that participants’ acceptability of NeW-I can be attributed to the cultural sensitivity of this intervention which makes it suitable for its target population—Asian parent-caregivers. Lastly, this study offers invaluable recommendations for future trials of NeW-I. For instance, future NeW-I studies would benefit from incorporating follow-up sessions over a longer interval (for instance, bi-monthly sessions), similar to other interventions [48–51]. Such follow-up sessions may invite parents to share their experiences of caregiving since their previous engagement with the NeW-I therapist, review the ideas that were discussed during the original NeW-I sessions and reflect on whether and how the knowledge and skills acquired during the NeW-I sessions were practiced in their daily life.

### Limitations and future directions

Readers are cautioned about the small and heterogeneous nature of the sample in the present study and the delivery of the intervention by a single therapist alone – both these attributes may limit the extent to which the findings can be generalized to other contexts and populations. Further, interviews for the acceptability study were carried out by the therapist who delivered the intervention. It is possible that participants’ responses may have been influenced by a perceived power dynamic between themselves and the therapist despite assurances that participants’ honest and heartfelt responses would be respected and used for enhancing future iterations of NeW-I. Moreover, despite multiple team meetings to ensure research rigour in the analysis of qualitative data and inter-researcher consensus in the finalizing of themes, it could be that the emergent themes were impacted by the research team’s subjectivity. It is recommended that future research must employ a larger and more diverse participant pool, include parent-caregivers from other Asian communities besides Singapore, and engage an evaluation team whose members are not directly involved in delivering the intervention is necessary to determine the extent to which NeW-I is effective in improving Asian parent-caregivers’ well-being as well as potential challenges that therapists need to be prepared for in delivering the intervention.

## Conclusion

This study describes the initial piloting of NeW-I—a promising online anticipatory grief intervention for parent-caregivers of children with CLTIs—demonstrating that it is both acceptable and feasible in the Singapore context. Qualitative evidence indicates that participants found NeW-I to be helpful, particularly in improving their perception of social support. Further evidence from an ongoing definitive trial is needed to assess its efficacy.

## Data Availability

All data collected during this study which can be made publicly available is included in this article. Additional data from the current study is not publicly available in order to safeguard the anonymity and confidentiality of participants. Such data may be available from the corresponding author on reasonable request.

## Abbreviations

CLTI: Chronic Life-Threatening Illness
NeW-I: Narrative e-Writing Intervention
PPC: Paediatric Palliative Care

## References

[1] Cunningham RM, Walton MA, Carter PM. The major causes of death in children and adolescents in the United States. N Engl J Med. 2018;379(25):2468–75. Available from: http://dx.doi.org/10.1056/NEJMsr1804754 10.1056/NEJMsr1804754 PMC6637963 30575483

[2] Fraser LK, Miller M, Hain R, Norman P, Aldridge J, McKinney PA, et al. Rising national prevalence of life-limiting conditions in children in England. Paediatrics. 2012 Apr [cited 2019 May 29];129(4):e923–9. Available from: http://dx.doi.org/10.1542/peds.2011-2846 10.1542/peds.2011-2846 22412035

[3] Connor S, Sisimayi C, Downing J, King E, Lim Ah Ken P, Yates R, et al. Assessment of the need for palliative care for children in South Africa. Int J Palliat Nurs. 2014;20(3):130–4. Available from: http://dx.doi.org/10.12968/ijpn.2014.20.3.130 10.12968/ijpn.2014.20.3.130 24675539

[4] Committee on the Rights of the Child. Singapore’s fourth and fifth periodic report. United Nations Convention on the Rights of the Child. 2017; Available from: https://www.reach.gov.sg/participate/public-consultation/ministry-of-social-and-family-development/rehabilitation-and-protection-group/public-consultation-on-singapores-periodic-report-on-the-uncrc

[5] Cadell S, Kennedy K, Hemsworth D. Informing social work practice through research with parent caregivers of a child with a life-limiting illness. J Soc Work End-of-Life Palliat Care. 2012;8(4):356–81. Available from: http://dx.doi.org/10.1080/15524256.2012.732021 10.1080/15524256.2012.732021 23194170

[6] Doka KJ. Ethics, end-of-life decisions and grief. Mortality. 2005;10(1):83–90. Available from: 10.1080/13576270500031105

[7] Bergstraesser E, Inglin S, Hornung R, Landolt MA. Dyadic coping of parents after the death of a child. Death Stud. 2015;39(1–5):128–38. Available from: http://dx.doi.org/10.1080/07481187.2014.920434 10.1080/07481187.2014.920434 25204680

[8] Alam R, Barrera M, D’Agostino N, Nicholas DB, Schneiderman G. Bereavement experiences of mothers and fathers over time after the death of a child due to cancer. Death Stud. 2012;36(1):1–22. Available from: https://www.ncbi.nlm.nih.gov/pubmed/24567992 10.1080/07481187.2011.553312 24567992

[9] Jordan J, Price JE, Prior L. Disorder and disconnection: Parent experiences of liminality when caring for their dying child. Soc Health Illn. 2015;37(6):839–55. Available from: http://dx.doi.org/10.1111/1467-9566.12235 10.1111/1467-9566.12235 26216375

[10] Meert KL, Eggly S, Pollack M, Anand KJS, Zimmerman J, Carcillo J, et al. Parents’ perspectives on physician-parent communication near the time of a child's death in the paediatric intensive care unit. Paediatr Crit Care Med. 2008;9(1):2–7. Available from: http://dx.doi.org/10.1097/01.PCC.0000298644.13882.88 10.1097/01.PCC.0000298644.13882.88 PMC3198033 18477906

[11] Dellve L, Samuelsson L, Tallborn A, Fasth A, Hallberg LR-M. Stress and well-being among parents of children with rare diseases: a prospective intervention study. J Adv Nurs. 2006;53(4):392–402. Available from: http://dx.doi.org/10.1111/j.1365-2648.2006.03736.x 10.1111/j.1365-2648.2006.03736.x 16448482

[12] Liben S, Papadatou D, Wolfe J. Paediatric palliative care: Challenges and emerging ideas. Lancet. 2008;371(9615):852–64. Available from: http://dx.doi.org/10.1016/S0140-6736(07)61203-3 10.1016/S0140-6736(07)61203-3 17707080

[13] Contro N, Sourkes BM. Opportunities for quality improvement in bereavement care at a children’s hospital: assessment of interdisciplinary staff perspectives. J Palliat Care. 2012 Spring;28(1):28–35. Available from: https://www.ncbi.nlm.nih.gov/pubmed/22582469 22582469

[14] Endo K, Yonemoto N, Yamada M. Interventions for bereaved parents following a child’s death: A systematic review. Palliat Med. 2015;29(7):590–604. Available from: http://dx.doi.org/10.1177/0269216315576674 10.1177/0269216315576674 25805741

[15] Dutta O, Tan-Ho G, Choo PY, Low XC, Chong PH, Ng C, et al. Trauma to transformation: the lived experience of bereaved parents of children with chronic life-threatening illnesses in Singapore. BMC Palliat Care. 2020;19(1):46. Available from: http://dx.doi.org/10.1186/s12904-020-00555-8 10.1186/s12904-020-00555-8 PMC7137327 32252753

[16] Dutta O, Tan-Ho G, Choo PY, Ho AHY. Lived experience of a child’s chronic illness and death: a qualitative systematic review of the parental bereavement trajectory. Death Stud. 2019;43(9):547–61. Available from: http://dx.doi.org/10.1080/07481187.2018.1503621 10.1080/07481187.2018.1503621 30285557

[17] Ho AHY, Dutta O, Tan-Ho G, Tan THB, Low CX, Ganapathy S, et al. A Novel Narrative E-Writing Intervention (NeW-I) for parents of children with chronic life-threatening illnesses: protocol for a pilot open-label randomized controlled trial. JMIR Res Protoc. 2020;9(7):e17561. Available from: http://dx.doi.org/10.2196/17561 10.2196/17561 PMC7380996 32623367

[18] Toyama H, Honda A. Using narrative approach for anticipatory grief among family caregivers at home. Glob Qual Nurs Res. 2016;3:1–15. Available from: http://dx.doi.org/10.1177/2333393616682549 10.1177/2333393616682549 PMC5342864 28462354

[19] Patinadan PV, Tan-Ho G, Choo PY, Ho AHY. Resolving anticipatory grief and enhancing dignity at the end-of life: a systematic review of palliative interventions. Death Stud. 2020 21;1–14. Available from: http://dx.doi.org/10.1080/07481187.2020.1728426 10.1080/07481187.2020.1728426 32079501

[20] Chochinov HM, Hack T, Hassard T, Kristjanson LJ, McClement S, Harlos M. Dignity therapy: a novel psychotherapeutic intervention for patients near the end of life. J Clin Oncol. 2005;23(24):5520–5. Available from: http://dx.doi.org/10.1200/JCO.2005.08.391 10.1200/JCO.2005.08.391 16110012

[21] Ho AHY, Car J, Ho M-HR, Tan-Ho G, Choo PY, Patinadan PV, et al. A novel Family Dignity Intervention (FDI) for enhancing and informing holistic palliative care in Asia: study protocol for a randomized controlled trial. Trials. 2017;18(1):587. Available from: http://dx.doi.org/10.1186/s13063-017-2325-5 10.1186/s13063-017-2325-5 PMC5715529 29202863

[22] Guo Q, Chochinov HM, McClement S, Thompson G, Hack T. Development and evaluation of the Dignity Talk question framework for palliative patients and their families: a mixed-methods study. Palliat Med. 2018;32(1):195–205. Available from: http://dx.doi.org/10.1177/0269216317734696 10.1177/0269216317734696 PMC5758936 29130367

[23] Julião M, Barbosa A, Oliveira F, Nunes B, Vaz Carneiro A. Efficacy of dignity therapy for depression and anxiety in terminally ill patients: early results of a randomized controlled trial. Palliat Support Care. 2013;11(6):481–9. Available from: http://dx.doi.org/10.1017/S1478951512000892 10.1017/S1478951512000892 23506744

[24] Julião M, Oliveira F, Nunes B, Vaz Carneiro A, Barbosa A. Efficacy of dignity therapy on depression and anxiety in Portuguese terminally ill patients: a phase II randomized controlled trial. J Palliat Med. 2014;17(6):688–95. Available from: http://dx.doi.org/10.1089/jpm.2013.0567 10.1089/jpm.2013.0567 24735024

[25] Vuksanovic D, Green HJ, Dyck M, Morrissey SA. Dignity therapy and life review for palliative care patients: a randomized controlled trial. J Pain Symptom Manage. 2017;53(2):162–70.e1. Available from: http://dx.doi.org/10.1016/j.jpainsymman.2016.09.005 10.1016/j.jpainsymman.2016.09.005 27810568

[26] Chochinov HM, Kristjanson LJ, Breitbart W, McClement S, Hack TF, Hassard T, et al. Effect of dignity therapy on distress and end-of-life experience in terminally ill patients: a randomised controlled trial. Lancet Oncol. 2011;12(8):753–62. Available from: http://dx.doi.org/10.1016/S1470-2045(11)70153-X 10.1016/S1470-2045(11)70153-X PMC3185066 21741309

[27] Ho AHY, Car J, Ho RMH, Tan-Ho G, Choo PY, Patinadan PV, et al. Family Dignity Intervention (FDI) for Advancing Asia Palliative Care: Preliminary findings from a Waitlist RCT. In: Association for Death Education and Counselling 41st Annual Conference. Atlanta, GA, USA; 11–13 April 2019. Available from: https://cdn.ymaws.com/www.adec.org/resource/resmgr/conference/2019_conference_abstract_boo.pdf

[28] Ho AHY, Car J, Ho M-HR, Tan-Ho G, Choo PY, Patinadan PV, et al. Family Dignity Intervention (FDI) for Advancing Asia Palliative Care: Preliminary findings from a Waitlist RCT. Association for Death Education and Counseling 41 st Annual Conference (Atlanta, GA, USA). 2019.

[29] Ho AHY, Car J, Ho RMH, Tan-Ho G, Choo PY, Patinadan PV, et al. Family Dignity Intervention (FDI) for advancing Holistic Care in Asia Palliative Care: Findings from a Randomized Controlled Trial. In: 13th Asia Pacific Hospice and Palliative Care Conference. Surabaya, Indonesia; 1–4 August 2019.

[30] Ho AHY. Living and dying with dignity: an interpretive-systemic framework in Hong Kong [Doctor of Philosophy]. Chan C, Chow AYM, editors. The University of Hong Kong; 2013. Available from: http://dx.doi.org/10.5353/th_b5106513

[31] Campbell M, Fitzpatrick R, Haines A, Kinmonth AL, Sandercock P, Spiegelhalter D, et al. Framework for design and evaluation of complex interventions to improve health. BMJ. 2000;321(7262):694–6. Available from: http://dx.doi.org/10.1136/bmj.321.7262.694 10.1136/bmj.321.7262.694 PMC1118564 10987780

[32] Lakshman R, Griffin S, Hardeman W, Schiff A, Kinmonth AL, Ong KK. Using the Medical Research Council framework for the development and evaluation of complex interventions in a theory-based infant feeding intervention to prevent childhood obesity: the baby milk intervention and trial. J Obes. 2014;2014:646504. Available from: http://dx.doi.org/10.1155/2014/646504 10.1155/2014/646504 PMC4131118 25143830

[33] Neimeyer RA. Bereavement and the quest for meaning: Rewriting stories of loss and grief. Hellenic J Psychol. 2006;3(3):181–8. Available from: https://pdfs.semanticscholar.org/34bc/cf1083c1d384ebf41612a6f51819b7c75c41.pdf

[34] McClement S, Chochinov HM, Hack T, Hassard T, Kristjanson LJ, Harlos M. Dignity therapy: Family member perspectives. J Palliat Med. 2007;10(5):1076–82. Available from: http://dx.doi.org/10.1089/jpm.2007.0002 10.1089/jpm.2007.0002 17985964

[35] Dutta O. Understanding Lived Experiences of Bereaved Parents of Children with Chronic Life-Threatening Illness: Towards a Culture-Specific and Meaning-Oriented Narrative E-Writing Intervention (NeW-I) for Anticipatory Grief Support in Singapore [Doctor of Philosophy]. Ho AHY, editor. Nanyang Technological University; 2020. Available from: http://dx.doi.org/10.32657/10356/144741

[36] Government of Singapore. Children and Young Persons Act. Singapore Statutes Online. 2019. Available from: https://sso.agc.gov.sg/Act/CYPA1993

[37] Club Rainbow Singapore. Club Rainbow (Singapore). 2015 [cited 2019 Jun 18]. Available from: https://www.clubrainbow.org/

[38] HCA Hospice Care. StarPALS (Paediatric Advanced Life Support): Collaborative Care for Children. 2017 [cited 2019 Jun 18]. Available from: https://www.hca.org.sg/Star-PALS

[39] Andrews G, Slade T. Interpreting scores on the Kessler Psychological Distress Scale (K10). Aust N Z J Public Health. 2001;25(6):494–7. Available from: http://dx.doi.org/10.1111/j.1467-842x.2001.tb00310.x 10.1111/j.1467-842x.2001.tb00310.x 11824981

[40] Kroenke K, Spitzer RL, Williams JB. The PHQ-9: Validity of a brief depression severity measure. J Gen Intern Med. 2001;16(9):606–13. Available from: http://dx.doi.org/10.1046/j.1525-1497.2001.016009606.x 10.1046/j.1525-1497.2001.016009606.x PMC1495268 11556941

[41] QSR International. NVivo. 2022 [cited 2022 Jan 13]. Available from: https://www.qsrinternational.com/nvivo-qualitative-data-analysis-software/home/

[42] Gale NK, Heath G, Cameron E, Rashid S, Redwood S. Using the framework method for the analysis of qualitative data in multi-disciplinary health research. BMC Med Res Methodol. 2013;13:117. Available from: https://bmcmedresmethodol.biomedcentral.com/articles/10.1186/1471-2288-13-117. 10.1186/1471-2288-13-117 PMC3848812 24047204

[43] Proctor E, Silmere H, Raghavan R, Hovmand P, Aarons G, Bunger A, et al. Outcomes for implementation research: conceptual distinctions, measurement challenges, and research agenda. Adm Policy Ment Health. 2011;38(2):65–76. Available from: http://dx.doi.org/10.1007/s10488-010-0319-7 10.1007/s10488-010-0319-7 PMC3068522 20957426

[44] Hedtke L. Creating Stories of Hope: A narrative approach to illness, death and grief. Aust N Z J Fam Ther. 2014;35(1):4–19. Available from: http://doi.wiley.com/10.1002/anzf.1040

[45] Rini A, Loriz L. Anticipatory mourning in parents with a child who dies while hospitalized. J Pediatr Nurs. 2007;22(4):272–82. Available from: http://dx.doi.org/10.1016/j.pedn.2006.08.008 10.1016/j.pedn.2006.08.008 17645955

[46] Ho AHY, Leung PPY, Tse DMW, Pang SMC, Chochinov HM, Neimeyer RA, et al. Dignity amidst liminality: healing within suffering among Chinese terminal cancer patients. Death Stud. 2013;37(10):953–70. Available from: http://dx.doi.org/10.1080/07481187.2012.703078 10.1080/07481187.2012.703078 24517523

[47] Ho AHY, Dutta O, Tan-Ho G, Choo PY, Low XC, Chong PH, et al. Thematic analysis of spousal interaction patterns among Asian parents of children with chronic life-threatening illness. BMJ Open. 2019 [cited 2019 Nov 20];9(11). Available from: https://bmjopen.bmj.com/content/9/11/e032582.full 10.1136/bmjopen-2019-032582 PMC6887006 31748309

[48] Gearing RE, Schwalbe CSJ, Lee R, Hoagwood KE. The effectiveness of booster sessions in CBT treatment for child and adolescent mood and anxiety disorders. Depress Anxiety. 2013;30(9):800–8. Available from: http://dx.doi.org/10.1002/da.22118 10.1002/da.22118 23596102

[49] Moos R, Schaefer J, Andrassy J, Moos B. Outpatient mental health care, self-help groups, and patients’ one-year treatment outcomes. J Clin Psychol. 2001;57(3):273–87. Available from: http://dx.doi.org/10.1002/jclp.1011 10.1002/jclp.1011 11241359

[50] Whisman MA. The efficacy of booster maintenance sessions in behavior therapy: review and methodological critique. Clin Psychol Rev. 1990;10(2):155–70. Available from: http://dx.doi.org/10.1016/0272-7358(90)90055-F

[51] Heber E, Lehr D, Ebert DD, Berking M, Riper H. Web-based and mobile stress management intervention for employees: a randomized controlled trial. J Med Internet Res. 2016;18(1):e21. Available from: http://dx.doi.org/10.2196/jmir.5112 10.2196/jmir.5112 PMC4749847 26818683

